# Assessment of Potential Drug–Drug Interactions for Novel Oral Melanocortin‐1 Receptor Agonist Dersimelagon

**DOI:** 10.1002/prp2.70069

**Published:** 2025-01-31

**Authors:** Akihito Ogasawara, Ryosuke Ide, Shinsuke Inoue, Minoru Tsuda, Renli Teng

**Affiliations:** ^1^ Mitsubishi Tanabe Pharma Corporation Tokyo Japan; ^2^ CareCeutics LLC Berwyn Pennsylvania USA

## Abstract

Dersimelagon is a novel investigational orally administered selective agonist of the melanocortin‐1 receptor. The drug–drug interaction (DDI) potential of dersimelagon was investigated in **both** nonclinical (in vitro) and clinical studies. The in vitro inhibition of CYP/UGT isoforms and efflux/uptake transporters by dersimelagon was assessed. The impact of 300‐mg dersimelagon on the pharmacokinetics (PK) of substrate drugs and the effect of co‐administering verapamil on 100‐mg dersimelagon PK (as substrate drug) were investigated in healthy participants in a Phase 1 study. DDIs were assessed based on ratios of *C*_max_ and AUC_0‐∞_ of substrate drug administered alone and with dersimelagon (or verapamil). Relatively potent in vitro inhibition of CYP2C9, CYP3A, UGT1A1, BCRP, P‐gp, and OATPs by dersimelagon was observed. In the clinical study, exposures of atorvastatin (CYP3A, P‐gp, BCRP, OATP substrate) rosuvastatin (BCRP and OATP substrate), and β**‐**hydroxy simvastatin (metabolite of simvastatin) increased 2‐ to 3‐fold (atorvastatin: C_max_ LS mean ratio = 198.0%; AUC_0‐∞_ ratio = 196.6%; rosuvastatin: *C*_max_ ratio = 316.5%, AUC_0‐∞_ ratio = 206.0%) when co‐administered with dersimelagon. Midazolam (CYP3A substrate), digoxin (P‐gp), pravastatin (OATP), and simvastatin (CYP3A) did not show any clinically relevant DDI effects when co‐administered with dersimelagon. Dersimelagon exposure increased ~25% when co‐administered with verapamil, an effect not considered clinically relevant. Dersimelagon 300 mg did not elicit major DDIs involving CYP/UGT enzymes and drug transporters; however, dersimelagon may have potential for clinically relevant DDIs with drugs that are substrates for BCRP, such as atorvastatin and rosuvastatin, and caution should be exercised when co‐administering 300‐mg dersimelagon with these statin drugs.

**Trial Registration:** ClinicalTrials.gov: NCT04793295, NCT04402489, NCT04440592, NCT02834442, NCT03520036, NCT03503266

## Graphical abstract

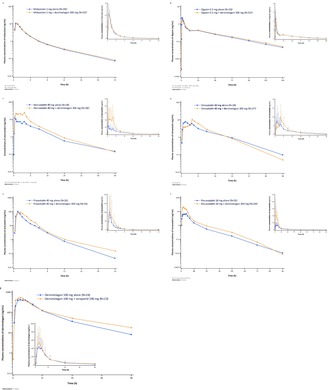

## 1 Introduction

Dersimelagon (formerly known as MT‐7117) is a novel orally administered synthetic nonpeptide small molecule selective agonist for melanocortin‐1 receptor that increases skin melanin without sun exposure [1]. Dersimelagon has recently been investigated in a phase 3 clinical trial (ClinicalTrials.gov ID NCT04402489) as a therapeutic option to increase light tolerance for patients with erythropoietic protoporphyria (EPP) and X‐linked protoporphyria (XLP) [2]. EPP and XLP are rare inherited photodermatoses characterized by acute, painful, nonblistering phototoxicity after prolonged sunlight exposure [3, 4]. Dersimelagon is also being evaluated (ClinicalTrials.gov ID NCT04440592) for use in diffuse cutaneous systemic sclerosis.

An earlier first‐in‐human phase 1 study (ClinicalTrials.gov ID NCT02834442) showed an acceptable safety profile for dersimelagon single ascending doses of 1 to 600 mg and multiple ascending doses of 30 to 450 mg in healthy adults [5]. In a phase 2, randomized, multicenter, placebo‐controlled clinical trial, ENDEAVOR (ClinicalTrials.gov ID NCT03520036), the safety and efficacy of 100‐mg and 300‐mg dersimelagon were investigated in patients with EPP or XLP [4, 6, 7]. In ENDEAVOR, dersimelagon was effective at increasing symptom‐free light exposure time, and had an acceptable safety and tolerability profile after 16 weeks of treatment [4, 6].

In a mass balance clinical study in healthy adults (ClinicalTrials.gov ID NCT03503266), rapid absorption and elimination were observed following oral administration of [^14^C] dersimelagon [8]. The primary route of excretion was feces (with a minor amount excreted in urine), and dersimelagon‐related components were not retained in tissues and organs. Unchanged dersimelagon was the main component in human plasma, and dersimelagon was extensively metabolized to the glucuronide in the liver, which was eliminated in bile and hydrolyzed to unchanged dersimelagon in the gut [8].

Drug–drug interaction (DDI) studies are integral components of the clinical drug development process, which are imperative to demonstrate whether a clinically relevant change in the exposure of a concomitantly administered drug alters the efficacy or safety profile of the other drug [9]. Cytochrome P450 (CYP) enzymes play a key role in drug disposition and DDIs by metabolizing many diverse drugs, while efflux/uptake transporters mediate the transport of various drugs across cell membranes [10]. The inhibition and induction of CYPs and transporters can significantly affect the toxicities and efficacies of their substrate drugs [10]. The results of DDI studies also can help guide clinicians in dose adjustments for the safe use of medications [9].

Preclinical in vitro studies and a phase 1 DDI study were conducted to evaluate whether dersimelagon is a clinically significant substrate, inhibitor, or inducer for relevant drug‐metabolizing enzymes and transporter proteins that are commonly implicated in DDIs. The effects of dersimelagon as a perpetrator drug on the pharmacokinetic (PK) profiles of substrate drugs, including midazolam (CYP3A probe substrate), digoxin (P‐glycoprotein [P‐gp]), atorvastatin (CYP3A, P‐gp, breast cancer resistance protein [BCRP], and organic anion transporting peptide [OATP] 1B1/1B3), simvastatin (CYP3A), pravastatin (OATP1B1/1B3), and rosuvastatin (BCRP, OATP1B1/1B3), were assessed. Additionally, the effect of verapamil (P‐gp inhibitor) as a perpetrator drug on the PK profile of dersimelagon as a substrate drug was investigated. The present findings will be used to assess the potential DDI effects of dersimelagon with commonly used drugs to guide the clinical use of dersimelagon, including dose adjustment guidance if needed.

## 2 Methods

### 2.1 In Vitro Studies

#### 2.1.1 Test Compounds and Materials

Unlabeled dersimelagon (MT‐7117) was synthesized at Mitsubishi Tanabe Pharma Corporation (Japan), and [^14^C]dersimelagon was synthesized by Sekisui Medical Corporation (Japan).

#### 2.1.2 Identification of UDP‐Glucuronosyltransferase (UGT) Isoforms Involved in the Metabolism of Dersimelagon

To clarify the UGT enzymes involved in the metabolism of dersimelagon free base, the metabolisms of [^14^C]dersimelagon free base in human liver microsomes and recombinant human UGT‐expressing microsomes were examined. Further details are provided in the Supporting Information Methods.

#### 2.1.3 Inhibitory Potential of Dersimelagon for CYP and UGT Isoforms

The inhibitory effects (direct and time dependent) of dersimelagon on the activities of CYP isoforms (CYP1A2, CYP2B6, CYP2C8, CYP2C9, CYP2C19, CYP2D6, and CYP3A) were examined. Briefly, each CYP substrate was incubated in the absence or presence of dersimelagon at concentrations of 0.1–100 μM in human liver microsomes, and parallel incubations with standard inhibitors for each enzyme were performed as positive controls (Table 1). The time‐dependent inhibition of CYP3A by dersimelagon was also evaluated in human liver microsomes pre‐incubated for 30 min with dersimelagon (0.1–100 μM) in the presence of nicotinamide adenine dinucleotide phosphate (NADPH). The inhibition of human UGT1A1, UGT1A3, and UGT2B7 by dersimelagon was investigated by assessing the glucuronidation of UGT‐selective substrates in human liver microsomes (Table 1). Full details of the in vitro methods are provided in the Supporting Information Methods.

**TABLE 1:** In vitro analysis of CYP and UGT isoform inhibitions by dersimelagon in human liver microsomes.

	Substrate	IC_50_ (μM)		Parameters		
		Direct inhibition	Time‐dependent inhibition	*K*_i_ (μM)	*K*_I_ (μM)	*k*_inact_ (min^−1^)
**CYP isoform**						
1A2	Phenacetin	> 100	72.2	N/A	N/A	N/A
2B6	Bupropion	48.9	36.3	N/A	N/A	N/A
2C8	Paclitaxel	24.2	13.9	N/A	N/A	N/A
2C9	Diclofenac	9.16	8.30	5.13	N/A	N/A
2C19	(*S*)‐Mephenytoin	23.4	28.1	N/A	N/A	N/A
2D6	Bufuralol	> 100	> 100	N/A	N/A	N/A
3A4	Midazolam	74.6	23.1	N/A	64.1	0.0141
3A4	Testosterone	89.5	30.6	N/A	N/A.	N/A
**UGT isoform**						
1A1	β‐Estradiol	1.45	N/A	1.19	N/A	N/A
1A3	Chenodeoxycholic acid	22.6	N/A	N/A	N/A	N/A
2B7	3′‐Azido‐3′‐deoxythymidine	> 50	N/A	N/A	N/A	N/A

Abbreviations: CYP, cytochrome P450; IC_50_, half maximal inhibitory concentration; *K*_I_, concentration yielding inactivation rate constant at the 1/2 k_inact_; *K*_i_, inhibition constant; *k*_inact_, maximum inactivation rate constant; N/A, not applicable; UGT, UDP‐glucuronosyltransferase.

#### 2.1.4 Induction Potential of Dersimelagon for CYP Isoforms

The inductive effects of dersimelagon on the mRNA expression levels of CYPs (CYP1A2, CYP2B6, and CYP3A4) were examined using cultured human hepatocytes. These mRNA expression levels were measured after exposure of dersimelagon (0.1, 0.3, 1, 3, 10, 30, and 50 μM) for 72 h in human hepatocytes obtained from three batches. Additional details are provided in the Supporting Information Methods.

#### 2.1.5 Inhibitory Potential of Dersimelagon for Drug Transporters

Inhibition of the efflux transporters P‐gp and BCRP by dersimelagon was conducted in Caco‐2 cell monolayers (Table 2). The inhibitory effects of dersimelagon on OATP1B1‐ or OATP1B3‐mediated uptake of each probe substrate (estradiol 17β‐D‐glucuronide) were investigated in human embryonic kidney 293 (HEK293) cells expressing human OATP1B1 or OATP1B3. An in vitro study of organic anion transporter (OAT)1, OAT3, organic cation transporter (OCT)2, multidrug and toxin extrusion (MATE) 1, and MATE2‐K inhibitions by dersimelagon was performed in HEK293 cells expressing human forms of these transporters. Additional details of the in vitro methods are provided in the Supporting Information Methods.

**TABLE 2:** In vitro analysis of drug transporter inhibition by dersimelagon.

Transporter	Category	Substrate	IC_50_ (μM)
P‐gp	Efflux transporter	[^3^H]Digoxin	0.349
BCRP	Efflux transporter	[^3^H]Estrone sulfate	0.467
OATP1B1	Hepatic transporter	[^3^H]Estradiol 17β‐D‐glucuronide	0.158
OATP1B3	Hepatic transporter	[^3^H]Estradiol 17β‐D‐glucuronide	0.0471
OAT1	Renal transporter	[^3^H]p‐Aminohippuric acid	71.2
OAT3	Renal transporter	[^14^C]Estrone sulfate	0.227
OCT2	Renal transporter	[^14^C]Metformin	> 28.7
MATE1	Renal transporter	[^14^C]Metformin	5.64
MATE2‐K	Renal transporter	[^14^C]Metformin	> 27.4

Abbreviations: BCRP, breast cancer resistance protein; IC_50_, half maximal inhibitory concentration; MATE, multidrug and toxin extrusion; OAT, organic anion transporting; OCT, organic cation transporter; P‐gp, P‐glycoprotein.

### 2.2 Clinical Study

#### 2.2.1 Ethics

The study protocols were reviewed and approved by the relevant Institutional Review Boards and regulatory authorities before implementing the study, and written informed consent was obtained from all participants before any assessment was performed. The trial was designed and conducted in accordance with the International Conference on Harmonization Harmonized Tripartite Guidelines for Good Clinical Practice, with applicable regional and local legislation (standard operating procedures in place at Mitsubishi Tanabe Pharma America Inc.), and with the ethical principles specified in the Declaration of Helsinki.

#### 2.2.2 Study Design

**FIGURE 1:**
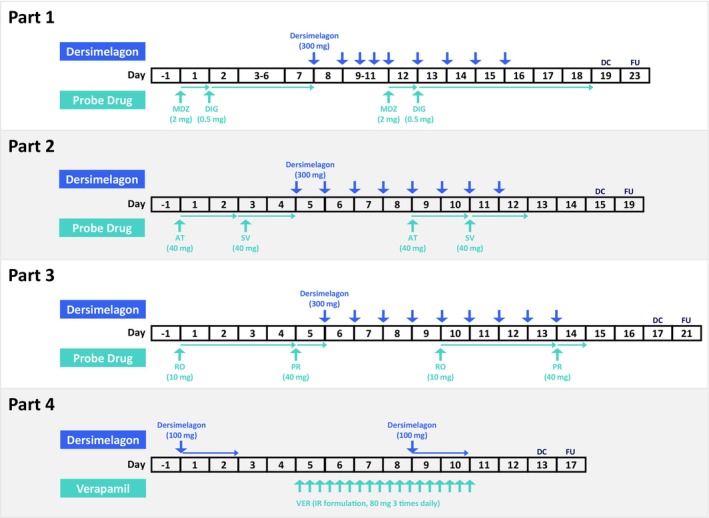
Study design schema for phase 1 clinical study for assessment of drug–drug interactions of dersimelagon. AT, atorvastatin; DC, discharge; DIG, digoxin; FU, follow‐up; IR, immediate‐release; MDZ, midazolam; PR, pravastatin; RO, rosuvastatin; SV, simvastatin; VER, verapamil.

This trial (NCT04793295) was conducted as a phase 1 multicenter, open‐label, four‐part, single‐sequence study in healthy adult participants to evaluate the effect of dersimelagon as a perpetrator of DDI with midazolam (CYP3A probe substrate), digoxin (P‐gp substrate), and statins (atorvastatin [CYP3A, P‐gp, BCRP, and OATP1B1/1B3 substrate], simvastatin [CYP3A substrate], pravastatin [OATP1B1/1B3 substrate], and rosuvastatin [BCRP and OATP1B1/1B3 substrate]) and as a victim of DDI with verapamil (Figure 1). The study methods are described in detail in the Supporting Information Methods.

#### 2.2.3 Study Population

The study enrolled healthy male and female participants aged 18–55 years and weighing at least 50 kg (110 pounds) with a body mass index of 18–30 kg/m^2^. Participants were instructed in restrictions on alcohol use, caffeine intake, smoking, and diet.

#### 2.2.4 Study Treatments

The test drugs used in the study (midazolam, digoxin, atorvastatin, simvastatin, pravastatin, rosuvastatin, and verapamil) are commonly used probe drugs in DDI studies and were selected from approved and marketed medications based on the metabolizing enzymes and transporters involved in their PK and inhibitory profiles. Standard commonly used doses (based on product labels) of reference drugs were administered in this study (Figure 1).

Participants in parts 1, 2 and 3 received a single dose of each of the substrate drugs (Figure 1). This was followed by 8–9 days of dersimelagon administration, with single doses of the substrate drugs also administered during that period. The substrate drugs in part 1 were digoxin and midazolam; in part 2, atorvastatin and simvastatin; and in part 3, pravastatin and rosuvastatin. In part 4, verapamil was administered on Days 5–10, and dersimelagon (as substrate drug) was administered on Days 1 and 9 (Figure 1).

In parts 1, 2 and 3, the planned maximum clinical dose of 300 mg dersimelagon (in ongoing trials) was considered appropriate for observing a maximum effect of dersimelagon on the PK profiles of the substrate drugs. A single dose of 100‐mg dersimelagon was used for part 4 because this dose was expected to elicit the maximum effect of verapamil (as a P‐gp inhibitor in the intestinal tract [data on file]) on the PK profile of dersimelagon. Dersimelagon doses from 100 to 300 mg daily are anticipated to be assessed for efficacy in phase 2 and phase 3 clinical trials. In parts 1–3, dersimelagon was administered for at least 5 days to achieve steady‐state plasma concentrations based on the results for multiple ascending doses in the first‐in‐human phase 1 study [5].

#### 2.2.5 PK Analyses

Blood samples were obtained according to schedules defined for each study part (further details are provided in the Supporting Information Methods). The plasma concentrations of dersimelagon, midazolam and its metabolites (1‐hydroxy midazolam and 4‐hydroxy midazolam), digoxin, atorvastatin and its metabolites (o‐hydroxy atorvastatin and p‐hydroxy atorvastatin), simvastatin and its metabolite (β‐hydroxy simvastatin), pravastatin, and rosuvastatin were determined using validated high‐performance liquid chromatography coupled with tandem mass spectrometry. The PK parameters assessed included maximum observed plasma concentration (C_max_) and area under the plasma concentration–time curve from time zero to infinity (AUC_0‐∞_).

#### 2.2.6 Statistical Analysis

The PK parameters were calculated by noncompartmental analysis (Phoenix WinNonlin version 8.2, Certara, Princeton, NJ), and all statistical analyses were performed using SAS version 9.4. A linear mixed‐effects model was fitted to log‐transformed PK parameters (C_max_ and AUC_0‐∞_) for midazolam, digoxin, atorvastatin, simvastatin, pravastatin, and rosuvastatin (parts 1, 2, and 3) and dersimelagon (part 4) using SAS, including treatment as a fixed effect and individuals as a random effect. Summary statistics of AUC_0‐∞_ were calculated using the individual data from subjects whose extrapolated AUC (AUC%ex) was not more than 20%. The least squares (LS) mean ratios and their corresponding 90% CIs on the log scale were then back transformed to provide LS mean and 90% CIs for the ratios of PK parameters with and without dersimelagon treatment (parts 1, 2, and 3) or verapamil treatment (part 4). The LS mean ratios of AUC_0‐∞_ of substrate drugs with/without dersimelagon were calculated using individual AUC_0‐∞_ with AUC%ex < 20%. If the 90% CIs for the LS mean ratios of interest of C_max_ and AUC_0‐∞_ fell within the equivalence range of 80%–125%, no DDI with dersimelagon was concluded.

#### 2.2.7 Safety Evaluations

Safety assessments included adverse events (AEs), laboratory parameters, vital signs, electrocardiography (ECG) parameters, and physical examinations.

#### 2.2.8 Nomenclature of Targets and Ligands

Key protein targets and ligands in this article are hyperlinked to corresponding entries in http://www.guidetopharmacology.org, the common portal for data from the IUPHAR/BPS Guide to PHARMACOLOGY [11], and are permanently archived in the Concise Guide to PHARMACOLOGY 2023/24: G protein‐coupled receptors [12].

## 3 Results

### 3.1 In Vitro Studies

#### 3.1.1 Identification of UGT Isoforms Involved in the Metabolism of Dersimelagon

Regarding the depletion of [^14^C]dersimelagon free base, dersimelagon glucuronide was generated mainly by UGT1A1 and UGT1A3 and slightly by UGT1A8. Results suggested that UGT1A1 and UGT1A3 played major roles in the metabolism of dersimelagon.

#### 3.1.2 Inhibition of CYP and UGT Activity by Dersimelagon

Dersimelagon directly inhibited CYP2C9, with an inhibition concentration of 9.16 μM to achieve half maximal inhibitory concentration (IC_50_; Table 1) and exhibited potential for time‐dependent inhibition of CYP3A. Lower inhibition was observed for CYP2C19 and CYP2C8. No notable inhibition of other CYP enzymes was observed with dersimelagon.

In addition, dersimelagon showed potent inhibition of UGT1A1 (IC_50_ = 1.45 μM), with lower inhibition observed for UGT1A3 (IC_50_ = 22.6 μM) and no notable inhibitory effect on UGT2B7. Dersimelagon showed competitive inhibition for UGT1A1, with a calculated inhibition constant (*K*_i_) value of 1.19 μM (Table 1).

Results of the basic and mechanistic static model assessments of dersimelagon for the inhibition of CYP/UGT isoforms are shown in Supporting Information Results Table 1. The R1 value of dersimelagon for CYP2C9 was below the cutoff value of 1.02. For UGT1A1 inhibition, the R1 values of 300‐mg dersimelagon were more than the cutoff value of 1.02. For CYP3A inhibition, the R1 value was below the cutoff value of 1.02, but the R_1,gut_ value was more than the cutoff value of 10 (basic model); in the mechanistic static model, the area under the plasma concentration–time curve ratio (AUCR) was more than the cutoff value of 1.25.

#### 3.1.3 Induction of CYP Enzymes by Dersimelagon

Under conditions in which prototypical inducers such as omeprazole, phenobarbital, and rifampicin caused the expected inductive effect on mRNA expression levels of CYP1A2, CYP2B6, and CYP3A4, respectively, dersimelagon did not cause an increase in the CYP1A2, CYP2B6, or CYP3A4 mRNA expression levels. These results indicated that dersimelagon had no inductive effect on CYP1A2, CYP2B6, or CYP3A4.

#### 3.1.4 Inhibition of Transporter Proteins by Dersimelagon

Dersimelagon inhibited P‐gp, BCRP, OATP1B1, OATP1B3, OAT1, OAT3, and MATE (Table 2). Concentration‐dependent inhibitions were observed on the transport of digoxin (model substrate for P‐gp) by dersimelagon at 0, 0.0123, 0.0444, 0.2, 0.948, 6.43, 26.43, and 47.8 μM (concentrations corrected with the adsorption ratio), and the IC_50_ value was calculated to be 0.349 μM. Concentration‐dependent inhibitions were observed on the transport of estrone sulfate (model substrate for BCRP) by dersimelagon at 0, 0.0123, 0.0444, 0.2, 0.948, 6.43, 26.43, and 47.8 μM, and the IC_50_ value was calculated to be 0.467 μM.

Concentration‐dependent inhibitions by dersimelagon at 0, 0.0184, 0.0658, 0.193, 0.617, 2.21, 8.01, and 27.9 μM were observed in OATP1B1‐ and OATP1B3‐expressing cells, and the IC_50_ values were calculated to be 0.158 μM and 0.0471 μM, respectively (Table 2).

The DDI potential of dersimelagon due to transporter inhibition was assessed based on the IC_50_ values of each transporter and the estimated systemic exposure of dersimelagon at steady state or gastrointestinal concentration following oral administration of 300‐mg dersimelagon (Supporting Information Results Table 2). The index values of P‐gp, BCRP, OATP1B1, OATP1B3, and OAT3 inhibition by 300‐mg dersimelagon, calculated as the ratio of each dersimelagon concentration, such as gastrointestinal concentration (*I*_gut_), estimated maximum unbound concentration at the inlet to the liver (*I*_inlet,max,u_), or maximum unbound concentration of dersimelagon in plasma (*I*_max,u_), and the IC_50_, were not sufficient to exclude the potential in vivo DDI of dersimelagon with substrate drugs of these transporters.

### 3.2 Clinical Study

#### 3.2.1 Participant Disposition and Baseline Characteristics

Of the 112 participants enrolled in the study, 109 participants completed the study. Two participants (out of 34 participants) withdrew consent in part 1 because of an adverse event (AE) and protocol noncompliance, and 1 participant (out of 28 participants) withdrew consent in part 2. No participant discontinued or withdrew consent in part 3 (*n* = 26) or part 4 (*n* = 24). Patient characteristics are shown in Table 3.

**TABLE 3:** Demographics and baseline characteristics.

	Part 1 midazolam and digoxin (*N* = 34)	Part 2 atorvastatin and simvastatin (*N* = 28)	Part 3 pravastatin and rosuvastatin (*N* = 26)	Part 4 verapamil (*N* = 24)
Age (years), mean (SD)	36.1 (10.2)	34.6 (8.5)	39.3 (8.5)	42.6 (9.1)
**Sex, *n* (%)**				
Male	17 (50.0)	17 (60.7)	8 (30.8)	7 (29.2)
Female	17 (50.0)	11 (39.3)	18 (69.2)	17 (70.8)
BMI (kg/m^2^), mean (SD)	25.6 (3.0)	26.7 (2.3)	26.8 (2.3)	27.4 (2.1)
White, *n* (%)	34 (100)	28 (100)	26 (100)	24 (100)
Hispanic or Latino ethnicity, *n* (%)	23 (67.6)	23 (82.1)	26 (100)	24 (100)
Never smoked, *n* (%)	27 (79.4)	24 (85.7)	26 (100)	24 (100)
Never consumed alcohol, *n* (%)	27 (79.4)	22 (78.6)	26 (100)	24 (100)

Abbreviation: BMI, body mass index.

#### 3.2.2 Effect of Dersimelagon on Midazolam (Part 1)

**FIGURE 2:**
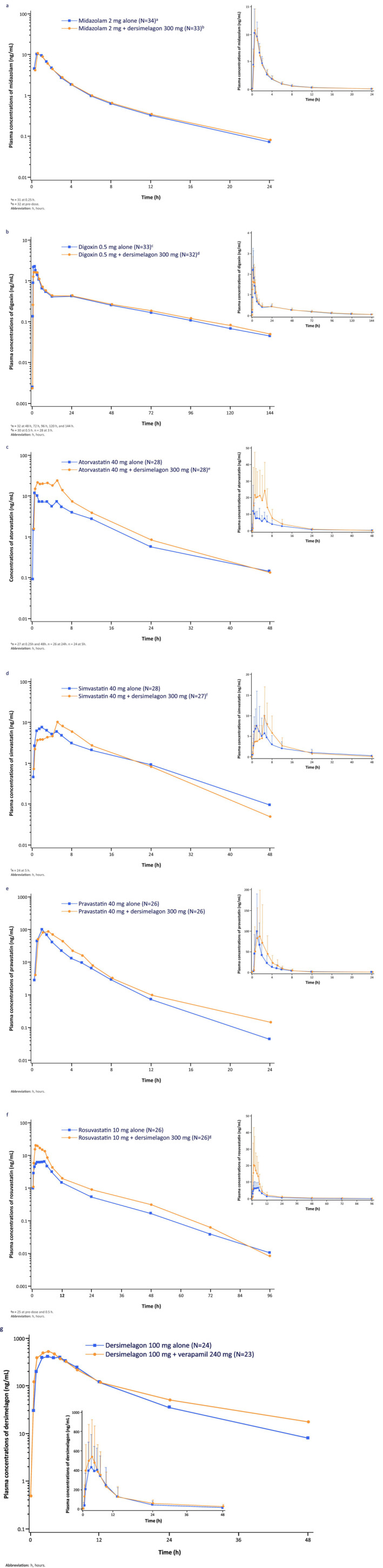
Mean plasma concentration–time profiles of (a) midazolam, (b) digoxin, (c) atorvastatin, (d) simvastatin, (e) pravastatin, and (f) rosuvastatin with and without dersimelagon and of (g) dersimelagon with and without verapamil on semi‐logarithmic scales (main figures) and linear scales (inserts). Data in inserts are displayed as mean + standard deviation. h, hours. ^a^ *n* = 31 at 0.25 h. ^b^ *n* = 32 at pre‐dose. ^c^ *n* = 32 at 48, 72, 96, 120, and 144 h. ^d^ *n* = 30 at 0.5 h. *n* = 28 at 3 h. ^e^ *n* = 27 at 0.25 h and 48 h. *n* = 26 at 24 h. *n* = 24 at 5 h. ^f^ *n* = 24 at 5 h. ^g^ *n* = 25 at pre‐dose and 0.5 h.

The mean plasma concentration–time curves of midazolam administered alone and co‐administered with dersimelagon are shown in Figure 2A. The overall exposure of plasma midazolam (C_max_ and AUC_0‐∞_) was comparable with and without co‐administration of dersimelagon, and the 90% CIs for the LS mean ratios for C_max_ and AUC_0‐∞_ fell within the equivalence range of 80%–125% (Table 4). For the midazolam metabolites 1‐hydroxy midazolam and 4‐hydroxy midazolam, the LS mean ratios for C_max_ were 123.3% and 106.9%, respectively, and AUC_0‐∞_ ratios were 125.7% and 111.2%, respectively (Supporting Information Results Table 3); the mean plasma concentration–time curves when administered alone and co‐administered with dersimelagon are shown in Supporting Information Results Figure 1a,b.

**TABLE 4:** Summary of pharmacokinetic parameters and drug–drug interaction effects (PK population).

Parameter	Midazolam (Part 1)		Digoxin (Part 1)		Atorvastatin (Part 2)		Simvastatin (Part 2)		Pravastatin (Part 3)		Rosuvastatin (Part 3)		Dersimelagon (Part 4)	
	Alone (*N* = 34)	+ Dersi (*N* = 33)	Alone (*N* = 33)	+ Dersi (*N* = 32)	Alone (*N* = 28)	+ Dersi (*N* = 28)	Alone (*N* = 28)	+ Dersi (*N* = 27)	Alone (*N* = 26)	+ Dersi (*N* = 26)	Alone (*N* = 26)	+ Dersi (*N* = 26)	Alone (*N* = 24)	+ Vera (*N* = 23)
***C***_**max**_														
Geometric mean (CV%)	10.66 (33.3)	10.70 (31.6)	2.45 (34.2)	2.02 (56.6)	14.95 (59.7)	29.59 (73.2)	8.08 (69.2)	8.56 (71.3)	76.35 (89.8)	76.79 (97.6)	6.59 (60.5)	20.88 (80.0)	450.7 (58.2)	558.7 (55.5)
LS mean	10.66	10.69	2.43	2.03	14.95	29.59	8.08	8.95	76.35	76.79	6.59	20.88	450.7	567.6
LS mean ratio (90% CI)	100.3 (93.5–107.6)		83.9 (72.7–96.7)		198.0 (160.7–243.8)		110.7 (93.7–130.8)		100.6 (75.7–133.7)		316.5 (265.1–377.9)		125.9 (107.4–147.7)	
**AUC**_**0‐∞**_														
Geometric mean (CV%)	28.1 (32.5)	28.0 (30.0)	37.4 (19.1)	37.9 (33.0)	85.3 (52.5)	168 (63.8)	62.2 (85.2)	75.9 (74.1)	160 (77.9)	210 (76.6)	64.2 (59.6)	128 (63.6)	4020 (66.7)	5150 (59.1)
LS mean	28.1	28.0	37.2	37.9	85.3	168.0	62.7	75.7	160.0	210.0	64.2	132.0	4020.0	5120.0
LS mean ratio (90% CI)	99.7 (93.7–106.1)		102.0 (94.1–110.5)		196.6 (176.6–218.7)		120.8 (97.9–148.9)		131.8 (109.0–159.2)		205.0 (181.2–231.8)		127.5 (116.0–140.1)	

*Note:* Data are presented as geometric mean (C_max_: ng/mL, AUC_0‐∞_: ng‧h/mL) (geometric CV%), LS mean (C_max_: ng/mL, AUC_0‐∞_: ng‧h/mL), and LS mean ratio (expressed as a percentage) (90% CI) with and without the perpetrator drug. The perpetrator drug is dersimelagon for parts 1–3 and verapamil for part 4. The doses used for each reference drug were 2, 0.5, 40, 40, 40, and 10 mg for midazolam, digoxin, atorvastatin, simvastatin, pravastatin, and rosuvastatin, respectively. Dersimelagon doses were 300 mg for parts 1–3 and 100 mg for part 4, and the verapamil dose was 240 mg for part 4.

Abbreviations: AUC_0‐∞_, area under the plasma concentration‐time curve from time zero to infinity; *C*_max_, maximum observed plasma concentration; CV, coefficient of variation; Dersi, dersimelagon; LS, least squares; PK, pharmacokinetic; Vera, verapamil.

#### 3.2.3 Effect of Dersimelagon on Digoxin (Part 1)

The mean plasma concentration–time curves of digoxin administered alone and with dersimelagon are shown in Figure 2b. While there was an approximate16% decrease observed for C_max_ of digoxin when administered with dersimelagon (LS mean ratio [90% CI]: 83.9% [72.7, 96.7]) there was no change to the AUC_0‐∞_ (102.0% [94.1, 110.5]; Table 4).

#### 3.2.4 Effect of Dersimelagon on Atorvastatin (Part 2)

The mean plasma concentration–time curves of atorvastatin administered alone and co‐administered with dersimelagon are shown in Figure 2c. Atorvastatin C_max_ and AUC_0‐∞_ showed an approximate 2‐fold increase following concomitant oral dosing of dersimelagon (Table 4). For the metabolites of atorvastatin (o‐hydroxy atorvastatin and p‐hydroxy atorvastatin), the LS mean ratios were increased 3‐ and 1.7‐fold, respectively, with concomitant dersimelagon (Supporting Information Results Table 3); the mean plasma concentration‐time curves when administered alone and co‐administered with dersimelagon are shown in Supporting Information Results Figure 1c,d.

#### 3.2.5 Effect of Dersimelagon on Simvastatin (Part 2)

The mean plasma concentration–time curves of simvastatin administered alone and with dersimelagon are shown in Figure 2d. The systemic exposure of simvastatin was slightly increased with co‐administration of dersimelagon (Table 4), with LS mean ratios for C_max_ and AUC_0‐∞_ of 110.7% and 120.8%, respectively. The upper limits of the corresponding 90% CIs did not fall within the equivalence range. For the metabolite of simvastatin (β‐hydroxy simvastatin), LS means for C_max_ and AUC0‐∞ were approximately 3‐ and 2‐fold higher, respectively, with concomitant dersimelagon (Supporting Information Results Table 3); the mean plasma concentration‐time curves when administered alone and co‐administered with dersimelagon are shown in Supporting Information Results Figure 1e.

#### 3.2.6 Effect of Dersimelagon on Pravastatin (Part 3)

The mean plasma concentration–time curves of pravastatin administered alone and co‐administered with dersimelagon are shown in Figure 2e. The systemic exposure to pravastatin indicated by LS mean ratio for C_max_ was equivalent with concomitant dersimelagon, but AUC0‐∞ was slightly higher (LS mean ratio = 131.8%; Table 4). Neither of the 90% CIs for these ratios fell within the equivalence range.

#### 3.2.7 Effect of Dersimelagon on Rosuvastatin (Part 3)

The mean plasma concentration–time curves of rosuvastatin administered alone and co‐administered with dersimelagon are shown in Figure 2f. The systemic exposure of rosuvastatin indicated by C_max_ and AUC_0‐∞_ showed a 2‐ to 3‐fold increase following concomitant dosing of dersimelagon (Table 4).

#### 3.2.8 PK Of Dersimelagon With and Without Verapamil (Part 4)

The mean plasma concentration–time curves of dersimelagon (as substrate drug) administered alone and co‐administered with verapamil are shown in Figure 2g. The dersimelagon C_max_ and AUC_0‐∞_ were approximately 25% higher after co‐administration of verapamil, and the 90% CIs around the LS mean ratios for both C_max_ and AUC_0‐∞_ fell outside the equivalence range (Table 4).

#### 3.2.9 Safety and Tolerability

In part 1 of the study, one participant experienced syncope after administration of midazolam and digoxin, which led to the participant discontinuing from the study. (This participant did not receive dersimelagon).

No serious AEs or serious adverse drug reactions were reported in any part of the study. The most common AEs in parts 1, 2, or 3 were skin and subcutaneous tissue disorders, including ephelides and skin hyperpigmentation, while the most common AEs reported in part 4 (such as headache and dizziness) were related to nervous system disorders most likely related to verapamil.

No clinically relevant changes were observed in physical examination, laboratory parameters, urinalysis, ECG, or vital signs in any part of the study.

## 4 Discussion

Dersimelagon is an investigational drug, and its safety profile in humans has not been fully investigated. As an agent intended for use in patients with EPP and XLP, it is important to evaluate the risk of clinically relevant DDIs of dersimelagon.

In the present study, the in vitro potential of dersimelagon to inhibit the major CYP/UGT isoforms and transporters was evaluated using pooled human liver microsomes, Caco‐2 cells, and transporter‐overexpressing cells. The findings from in vitro studies indicated inhibition potential of dersimelagon toward CYP2C9, CYP2C19, and CYP2C8, as well as time‐dependent inhibition of CYP3A. Of the CYP enzymes, the lowest IC_50_ values were observed for the inhibition of CYP2C9, indicating that this isoform is the most sensitive to inhibition by dersimelagon. Interestingly, dersimelagon did not show inductive effects on CYP1A2, CYP2B6, or CYP3A enzymes, as evidenced by the lack of increase observed in mRNA expression levels. Dersimelagon also showed a potent inhibition of UGT1A1 and lower inhibition of UGT1A3. Although there is a risk of in vivo DDI by UGT1A1 inhibition when co‐administered with dersimelagon, given the small magnitude of DDI mediated by UGT1A1 inhibition with the limited values of *C*_max,u_/*K*_i_: 0.04 (Supporting Information Results Table 1), the effect of dersimelagon on the PK profile of typical substrates of UGT1A1 was not assessed.

Dersimelagon showed the potential to inhibit the transporter proteins P‐gp, BCRP, OATP1B1, and OATP1B3, with IC_50_ values of 0.0471–0.467 μM. Additionally, in vitro data indicated that dersimelagon is a substrate of P‐gp, and the risk of DDI with drugs that inhibit P‐gp cannot be excluded based on the results from in vitro experiments (data not shown).

The risk of in vivo DDI due to OAT3 inhibition was not assessed in this study because the results of DDI simulation using the dynamic PK model (physiologically based PK model) of dersimelagon and methotrexate (typical OAT3 substrate) showed that DDIs are unlikely with concomitant administration of 300‐mg dersimelagon (data not shown). The potential of dersimelagon to inhibit CYP3A, P‐gp, BCRP, and OATPs in vivo cannot be excluded based on the results of the basic, mechanistic static, and dynamic model assessments of dersimelagon for inhibition of CYPs and UGTs and calculation of index values (e.g., *R* value or I_max,u_/IC_50_ value) for transporters that are recommended for evaluation in US Food and Drug Administration DDI guidance [13]. Therefore, the drug metabolizing enzyme‐ and transporter‐mediated DDI potential of concomitant administration of dersimelagon with CYP3A, P‐gp, BCRP, and OATP substrates was assessed in a phase 1 clinical study to investigate the DDI potential of dersimelagon.

In the DDI clinical study, systemic plasma exposure of midazolam and digoxin were comparable with and without co‐administration of 300‐mg dersimelagon, indicating no DDI effect with the CYP3A substrate midazolam and a minor DDI effect with the P‐gp substrate digoxin. In contrast, a 2‐ to 3‐fold increase in systemic exposure was observed for atorvastatin and rosuvastatin, both known to be BCRP and OATP1B1/1B3 substrates. Co‐administration with dersimelagon had a limited (1.1‐ and 1.2‐fold increase in the C_max_ and AUC_0‐∞_, respectively) effect on simvastatin (CYP3A substrate) exposure. On the other hand, the C_max_ and AUC_0‐∞_ of its metabolite, β‐hydroxy simvastatin (simvastatin acid; OATP1B1 substrate), was increased 2.9‐ and 2.2‐fold, respectively, when co‐administered with dersimelagon. The effect of dersimelagon on the plasma concentration of pravastatin (OATP1B1 substrate)—1.0‐fold and 1.3‐fold increases in C_max_ and AUC_0‐∞_, respectively—was relatively small compared with that for simvastatin acid. Considering the effect of OATP variants (c.521 T>C), which have been associated with changes in the in vitro transporter activity, on the PK profile of pravastatin and simvastatin acid [14, 15], OATP1B1 contributes more to the PK profile of simvastatin acid than pravastatin, suggesting that the significant effect of dersimelagon on simvastatin acid is due to OATP1B1 inhibition. The results of the in vivo DDI studies showed that dersimelagon had a greater effect on the systemic exposure of pravastatin (OATP1B1 substrate) but not midazolam (CYP3A substrate). Therefore, it is reasonable that dersimelagon increased the systemic exposure of simvastatin acid (OATP1B1 substrate) but did not affect the plasma concentration of simvastatin (CYP3A substrate). Notably, the impacts of dersimelagon on the PK profile of simvastatin and simvastatin acid are similar to that of the *SLCO1B1* (encodes OATP1B1) variant (c.521 T>C) on the PK profile of simvastatin and simvastatin acid [15]. Although the regulatory documents such as package insert for simvastatin does not define dose adjustment of simvastatin in patients with the SLCO1B1 variant, caution should be exercised when simvastatin and dersimelagon are used concomitantly.

In the in vitro vesicular transport assay, only 2‐hydroxyatorvastatin was taken up in BCRP vesicles significantly more than in control vesicles. The accumulation ratio for other metabolites, 4‐hydroxyatorvastatin and simvastatin acid, were not significantly different compared to control vesicles [16]. For the contribution of the ABC transporters to atorvastatin and rosuvastatin, BCRP was a major efflux transporter for rosuvastatin at the intestine and liver; however, BCRP appeared to have a limited role as an efflux transporter for atorvastatin in the intestine and liver. In contrast, P‐gp appeared to be the major efflux transporter for atorvastatin in the intestine and liver with a minor contribution to rosuvastatin PK. ABC transporters did not contribute significantly to the PK profiles of pravastatin and simvastatin acid. The large contribution of BCRP to the PK profile of rosuvastatin compared to that of atorvastatin was also reproduced in the PK study in subjects with the ABC subfamily G member 2 (ABCG2) polymorphism [17]. Thus, the various effects of dersimelagon on the PK profile of different statins in our study appears to be dependent on the contribution of each transporter to the PK of the statin.

In this clinical DDI study, plasma concentrations of the first substrate drug just before dosing of second substrate drug were below the limit of quantification, except for rosuvastatin. Rosuvastatin concentrations just before dosing of pravastatin (Part 3) were less than 5% of rosuvastatin C_max_. To our knowledge, there has been no report of the potential of rosuvastatin to inhibit the drug metabolizing enzymes and transporters. Additionally, DDI with dersimelagon had limited impact on the time to reach *C*_max_ (*T*_max_) of most substrate drugs (data not shown). Median T_max_ of simvastatin was delayed from 1.5 h to 5 h when co‐administered with dersimelagon. The interaction between simvastatin and dersimelagon during the gastrointestinal absorption process is unlikely based on the PK profile of simvastatin and the DDI profile of dersimelagon. Since the biotransformation of simvastatin to simvastatin acid is reversible, the elevated systemic exposure of simvastatin acid might be related to the delayed T_max_ of simvastatin, although the exact mechanism involved remains to be elucidated.

The preclinical in vitro data indicated that dersimelagon is likely to be a substrate for the efflux transporters P‐gp. Consequently, P‐gp inhibitors may be expected to alter the PK of dersimelagon. In the clinical DDI study, exposure of dersimelagon increased approximately 25% after co‐administration of verapamil, a P‐gp inhibitor. However, the effect of verapamil on the PK profile of dersimelagon was not considered clinically relevant and may have been limited because of the good membrane permeability of dersimelagon observed in the in vitro experiment using Caco‐2 cells.

In the present phase 1 clinical study in healthy individuals, dersimelagon was well tolerated when administered alone or in combination with oral doses of midazolam, digoxin, atorvastatin, simvastatin, pravastatin, rosuvastatin, and verapamil. There were no clinically relevant changes in the safety profiles of dersimelagon or the comedications when concomitantly administered. The most common AEs in parts 1, 2, and 3 were skin and subcutaneous tissue disorders, which is consistent with the clinical profile expected from the known mechanism of action for dersimelagon.

Collectively, the present in vitro and phase 1 clinical studies demonstrate that 300‐mg dersimelagon has a low potential for DDIs, except for interactions with BCRP substrates such as atorvastatin and rosuvastatin. Statins are drugs widely prescribed to treat hypercholesterolemia in the United States [18]; therefore, the present study findings provide important information that will help to establish prohibited medications or dose adjustments in clinical studies with patients with EPP, XLP, or other diseases. Furthermore, the observed PK data will be valuable in simulating the DDI potential of dersimelagon with the different dosing regimens used in this clinical DDI study in an ongoing physiologically based PK model analysis.

## 5 Conclusions

Results of the current in vitro studies indicate that the potential of dersimelagon to inhibit CYP3A, P‐gp, BCRP, and OATP activities in vivo cannot be excluded. In the phase 1 clinical study, mild to moderate DDI potential of 300‐mg dersimelagon with statins was observed, suggesting caution should be taken when co‐administering these drugs. In summary, the results of the in vitro and clinical PK studies of dersimelagon did not reveal major DDIs to be expected involving CYP/UGT enzymes and drug transporters, except for BCRP inhibition, which may require further investigation.

## Author Contributions

**Akihito Ogasawara:** conceptualization, methodology, formal analysis, project administration, writing – original draft. **Ryosuke Ide:** investigation, project administration, supervision. **Shinsuke Inoue:** formal analysis, project administration. **Minoru Tsuda:** formal analysis, investigation, project administration. **Renli Teng:** conceptualization, methodology, All authors: writing – reviewing and editing.

## Ethics Statement

The trial was designed and conducted in accordance with the International Conference on Harmonization Harmonized Tripartite Guidelines for Good Clinical Practice, with applicable regional and local legislation (standard operating procedures in place at Mitsubishi Tanabe Pharma America Inc.), and with the ethical principles specified in the Declaration of Helsinki.

## Consent

The study protocols were reviewed and approved by the relevant Investigational Review Board and regulatory authorities before implementing the study, and written informed consent was obtained from all participants before any assessment was performed.

## Conflicts of Interest

Akihito Ogasawara, Ryosuke Ide, and Minoru Tsuda are employees of Mitsubishi Tanabe Pharma Corporation. a partner of CareCeutics LLC.

## Supporting information


Data S1.

## Data Availability

Data may be made available upon reasonable request.
