# Supplementary material for: Assessment of Potential Drug–Drug Interactions for Novel Oral Melanocortin‐1 Receptor Agonist Dersimelagon
Source: Pharmacol Res Perspect. 2025 Jan 31;13(1):e70069. doi: 10.1002/prp2.70069 (PMC11783400; doi:10.1002/prp2.70069)

# SUPPLEMENTARY MATERIAL for

# Assessment of Potential Drug-Drug Interactions for Novel Oral Melanocortin-1 Receptor Agonist Dersimelagon

Ogasawara A, et al.

# Supplementary Methods

## In Vitro Studies

**Identification of UGT enzymes involved in the metabolism of [^14^C]dersimelagon free base**

To clarify the UDP-glucuronosyltransferase (UGT) enzymes involved in the metabolism of dersimelagon free base, the metabolisms of [^14^C]dersimelagon free base in human liver microsomes and recombinant human UGT-expressing microsomes fortified with UDPGA were examined.

First, as a result of metabolism of [^14^C]dersimelagon free base in human liver microsomes, dersimelagon glucuronide was found only as a metabolite of [^14^C]dersimelagon free base. As a result of metabolism in recombinant human UGT-expressing microsomes (UGT1A1, UGT1A3, UGT1A4, UGT1A6, UGT1A7, UGT1A8, UGT1A9, UGT1A10, UGT2B4, UGT2B7, UGT2B10, UGT2B15, and UGT2B17), [^14^C]dersimelagon free base (1 and 10 μM) was metabolized mainly by UGT1A1 and UGT1A3, and slightly by UGT1A8. Furthermore, in human liver microsomes, dersimelagon glucuronide was only found as a metabolite of [^14^C]dersimelagon free base in incubation with recombinant human UGT-expressing microsomes.

Regarding the depletion of [^14^C]dersimelagon free base described above, dersimelagon glucuronide was also generated mainly by UGT1A1 and UGT1A3, and slightly by UGT1A8. Formation velocity of dersimelagon glucuronide at 1 μM in the 30-minute and 60-minute incubation mixtures was 18.2 and 13.9 pmol/min/mg protein, respectively, for UGT1A1; 19.7 and 15.9 pmol/min/mg protein, respectively, for UGT1A3; and 2.37 and 2.20 pmol/min/mg protein, respectively, for UGT1A8.

At 10 μM, the formation velocity of dersimelagon glucuronide in the 30-min and 60-min incubation mixtures was 85.7 and 74.0 pmol/min/mg protein, respectively, for UGT1A1; 131 and 103 pmol/min/mg protein, respectively, for UGT1A3; and 3.67 and 4.17 pmol/min/mg protein, respectively, for UGT1A8.

From these results, it was suggested that UGT1A1 and UGT1A3 played major roles in the metabolism of dersimelagon free base.

**Inhibitory Effect of Dersimelagon on the Specific Activities of Human Cytochrome P450 (CYP) Isoforms**

Human liver microsomes (20 mg protein/mL) were diluted 10-fold with 0.1 mol/L Na-K phosphate buffer (pH 7.4) in an ice bath. The dilution was performed just before use, and the prepared microsome suspensions were kept in an ice bath until use. The half maximal inhibitory concentration (IC_50_) values of dersimelagon were determined on the specific metabolic activities of CYP isoforms without pre-incubation of dersimelagon in the presence of the nicotinamide adenine dinucleotide phosphate (NADPH) in human liver microsomes to examine the effects of direct inhibition of dersimelagon. In addition, to evaluate inactivation potencies of dersimelagon, the IC_50_ shift was examined with human liver microsomes pre-incubated with dersimelagon in the presence of the NADPH. Well-known specific inhibitors for each CYP isoform were used as the positive control, and the marker activities of CYP isoforms are listed here:

1. CYP1A2: Phenacetin *O*-deethylation
2. CYP2B6: Bupropion hydroxylation
3. CYP2C8: Paclitaxel 6α-hydroxylation
4. CYP2C9: Diclofenac 4'-hydroxylation
5. CYP2C19: (*S*)-Mephenytoin 4-hydroxylation
6. CYP2D6: Bufuralol 1'-hydroxylation
7. CYP3A: Midazolam 1'-hydroxylation
8. CYP3A: Testosterone 6α-hydroxylation

Incubation was performed by the following two methods:

**Direct inhibition method:** The reaction mixtures were pre-warmed at 37 °C for 5 minutes before addition of the NADPH solution. The reaction was initiated with addition of the NADPH solution and conducted for the designated time at 37 °C.

**Time-dependent inhibition method:** The reaction mixtures were pre-warmed at 37 °C for 5 minutes before addition of the NADPH solution and substrate. After addition of the NADPH solution, the reaction mixtures were pre-incubated at 37 °C for 30 minutes. The reaction was initiated with addition of the substrate solution and conducted for the designated time at 37 °C.

**Inhibitory Effect of Dersimelagon on UGTs in Human Liver Microsomes**

IC_50_ values of dersimelagon were determined for the specific metabolic activities of UGT isoforms in the presence of the uridine 5'-diphospho-glucuronic acid (UDPGA) in human liver microsomes to examine the effects of inhibition of dersimelagon. Well-known specific inhibitors for each UGT isoform were used as positive controls, and the marker activities of UGT isoforms are listed here:

1. UGT1A1: β-Estradiol 3-glucuronidation

2. UGT1A3: Chenodeoxycholic acid 24-glucuronidation

3. UGT2B7: 3'-Azido-3'-deoxythymidine β-D-glucuronidation

Incubation was performed by the following method. The reaction mixtures were pre-warmed at 37 °C for 5 minutes before addition of the UDPGA solution. The reaction was initiated with addition of the UDPGA solution and conducted for 10 minutes at 37 °C.

**Inductive Effect of Dersimelagon on CYP Isoforms**

The human hepatocyte suspensions were plated on a collagen-coated plate and pre-cultured in a CO_2_ incubator for 4 hours. After rinsing the hepatocytes, the culture medium containing dersimelagon or a prototypical inducer (omeprazole [CYP1A2], phenobarbital [CYP2B6], or rifampicin [CYP3A4]) was added, and the cells were cultured in a CO_2_ incubator for 72 hours. Total RNA was collected from the hepatocytes, and mRNAs encoding human CYP1A2, CYP2B6, and CYP3A4 were measured by quantitative reverse transcription-polymerase chain reaction.

**In Vitro Evaluation of Dersimelagon as an Inhibitor of P-Glycoprotein (P-gp) and Breast Cancer Resistance Protein (BCRP) in Caco-2 Cells**

Caco-2 cells were washed with Hank’s Balanced Salt Solution, N-(2-hydroxyethyl)piperazine-N′-(2-ethanesulfonic acid) (HBSS-HEPES), and the medium was replaced with each of the pre-incubation media and pre-incubated in a constant temperature incubator shaker at 37 °C at 35 rpm for 1 hour. The medium containing a probe substrate ([^3^H]digoxin [P-gp] or [^3^H]estrone sulfate [BCRP]) and dersimelagon in either the apical or basal side was replaced with each of the incubation media. The other side was replaced with fresh pre-incubation medium, and the cells were incubated in a constant temperature incubator shaker at 37 °C at 35 rpm. After incubation for 2 hours, the medium (apical: 50 μL, basal: 100 μL) was collected from the side to measure the radioactivity, to which the incubation medium (containing the model substrate or reference compound) was not added. From the obtained radioactivity, the permeability coefficient (P_app_) of apical to basal and basal to apical and efflux ratio were determined to assess the inhibitory effects. The result obtained from [^14^C]mannitol was used as an index for formation of the cell monolayer.

**In Vitro Evaluation of the Inhibitory Effects of Dersimelagon on the Uptake of Substrate Mediated by** **Organic Anion Transporting Peptide (OATP)1B1 and OATP1B3**

The culture medium was removed from plates seeded with transporter-expressing cells or control cells, and 1 mL of HBSS containing 0.2% dimethyl sulfoxide (DMSO) was added. After removal of HBSS from the plates, 300 μL of dersimelagon solution was added to the plates, and the plates were pre-incubated at 37 °C for 30 minutes. The preincubation solution was completely removed from the plates, and 300 μL of the reaction solutions containing [^3^H]estradiol 17β-D-glucuronide (probe substrate) and dersimelagon were added to the plates. The plates were incubated at 37 °C. After incubation for 2 minutes, the reaction solutions were removed from the plates, and cells were washed once with 1 mL of ice-cold phosphate-buffered saline (PBS) containing 0.2% bovine serum albumin (BSA) and twice with 1 mL of ice-cold PBS. After removal of PBS completely, the cells were dissolved in 0.5 mL of 0.1 mol/L NaOH. The cell lysates were mixed adequately by pipetting, and 300-μL aliquots of the cell lysates were collected in polypropylene vials. The scintillation cocktail (5 mL, Emulsifier-Safe; PerkinElmer Inc) was added to each vial, and the radioactivity was measured using a liquid scintillation counter.

**In Vitro Evaluation of the Inhibitory Effects of Dersimelagon on the Uptake of Substrate Mediated by OAT1, OAT3, OCT2, Multidrug and Toxin Extrusion (MATE) 1, and MATE2-K**

This was assessed using human transporter-expressing cells and control cells. The culture medium was removed from plates seeded with transporter-expressing cells or control cells, and
1 mL of HBSS (pH 7.4 or pH 8.5 [for MATE1 and MATE2-K]) was added. After removal of HBSS from the plates, 300 μL of HBSS (pH 7.4 or pH 8.5 (for MATE1 and MATE2-K) was newly added, and the plates were pre-incubated at 37 °C for 15 minutes. The pre-incubation solution was completely removed from the plates, and 300 μL of the reaction solutions containing a probe substrate ([^3^H]aminohippuric acid [OAT1], [^3^H]estrone sulfate [OAT3], or [^14^C]metformin [OCT2, MATE1, MATE2-K]) and dersimelagon were added to the plates. The plates were incubated at 37 °C. After incubation for the designated times, the reaction solutions were removed from the plates, and cells were washed once with 1 mL of ice-cold PBS containing 0.2% BSA and twice with 1 mL of ice-cold PBS. After removal of PBS completely, the cells were dissolved in 0.5 mL of 0.1 mol/L NaOH. The cell lysates were mixed adequately by pipetting, and 300-μL aliquots of the cell lysates were collected in polypropylene vials. The scintillation cocktail (5 mL, Emulsifier-Safe) was added to each vial, and the radioactivity was measured using a liquid scintillation counter.

## Phase 1 Clinical Study Design

**Study Population**

Participants with any concomitant or history of any hepatobiliary disease; neurological conditions; endocrine, thyroid, respiratory, gastrointestinal, renal, or cardiovascular disease; or any history (within the last 2 years) of any clinically significant psychiatric/psychotic illness disorder (including anxiety, depression, and reactive depression) were excluded from the study. Participants with any history of gastrointestinal/bariatric surgery (except uncomplicated surgical procedures such as appendectomy) also were excluded.

**Part 1: Midazolam and Digoxin**

Participants received a single oral dose of midazolam 2 mg on the morning of day 1 after an overnight fast, followed by a pharmacokinetic (PK) sampling period of 24 hours to measure plasma concentrations of midazolam, 1-hydroxy midazolam, and 4-hydroxy midazolam. Participants received a single oral dose of digoxin 0.5 mg on the morning of day 2 after an overnight fast, followed by blood sampling up to 144 hours postdose to determine the plasma concentration of digoxin. Dersimelagon 300 mg (3 ×100 mg tablets) was orally administered once daily from day 8 to day 16 with coadministration of midazolam 2 mg and digoxin 0.5 mg on day 12 and day 13, respectively. Blood samples to determine plasma concentrations of midazolam, midazolam metabolites, and digoxin were collected from day 12 to day 19 to evaluate the effect of dersimelagon on the PK profiles of midazolam, midazolam metabolites, and digoxin. For the determination of dersimelagon plasma concentrations, blood samples were collected just before daily dosing of dersimelagon on day 9 to day 11 and up to 24 hours postdose on day 12 and day 13.

To determine the concentrations of dersimelagon, plasma samples were prepared by solid phase extraction and analyzed using validated high-performance liquid chromatography coupled with tandem mass spectrometry (LC/MS/MS) with a lower limit of quantification (LLOQ) of 0.1 ng/mL. Deuterium-substituted free base of dersimelagon (D_6_-dersimelagon) was used as the internal standard. To determine the concentrations of midazolam, 1-hydroxy midazolam, and 4-hydroxy midazolam, plasma samples were prepared by liquid-liquid extraction and analyzed using a validated LC/MS/MS method with an LLOQ of 0.05, 0.2, and 0.01 ng/mL for midazolam, 1-hydroxy midazolam, and 4-hydroxy midazolam, respectively. Deuterium-substituted compounds of the analytes were used as the internal standard. To determine the concentrations of digoxin, plasma samples were prepared by liquid-liquid extraction and analyzed using a validated LC/MS/MS method with an LLOQ of 10 pg/mL. Deuterium-substituted compound of the analyte was used as the internal standard. In all cases, detection was performed using multiple reaction monitoring. Accuracy and coefficient of variation on sample analyses for all the analytes met the prespecified acceptance criteria.

**Part 2: Atorvastatin and Simvastatin**

Participants received a single oral dose of atorvastatin 40 mg on the morning of day 1 after an overnight fast, and blood samples were collected up to 48 hours postdose to determine plasma concentrations of atorvastatin and its active metabolites, o-hydroxy atorvastatin and p-hydroxy atorvastatin. The individuals received a single oral dose of simvastatin 40 mg on the morning of day 3 after an overnight fast, followed by blood sampling to determine plasma concentrations of simvastatin and its active metabolite, β**-**hydroxy simvastatin, up to 48 hours postdose. Dersimelagon 300 mg was administered once daily from day 5 to day 12 with coadministration of atorvastatin 40 mg and simvastatin 40 mg on day 9 and day 11, respectively. Blood samples were drawn from day 9 to day 13 to determine the plasma concentrations of atorvastatin, simvastatin, and their metabolites up to 48 hours postdose. To determine dersimelagon plasma concentrations, blood samples were collected just before daily dosing of dersimelagon from day 6 to day 8 and up to 24 hours postdose on day 9 and day 11.

The bioanalytical methods for dersimelagon were described in the methods for part 1. To determine the concentrations of atorvastatin, o-hydroxy atorvastatin, and p-hydroxy atorvastatin, plasma samples were prepared by liquid-liquid extraction and analyzed using a validated LC/MS/MS method with an LLOQ of 0.02, 0.0194, and 0.00913 ng/mL for atorvastatin, o-hydroxy atorvastatin, and p-hydroxy atorvastatin, respectively. To determine the concentrations of simvastatin and β**-**hydroxy simvastatin, plasma samples were prepared by solid phase extraction and analyzed using a validated LC/MS/MS method with an LLOQ of 0.1 ng/mL for simvastatin and β**-**hydroxy simvastatin. In all cases, deuterium-substituted compounds of the analytes were used as the internal standard. Detection was performed using multiple reaction monitoring. Accuracy and coefficient of variation on sample analyses for all analytes met the prespecified acceptance criteria.

**Part 3: Rosuvastatin and Pravastatin**

Participants received a single oral dose of rosuvastatin 10 mg on the morning of day 1 after an overnight fast, followed by a 96-hour blood sampling period postdose. Participants were administered a single oral dose of pravastatin 40 mg on the morning of day 5 after an overnight fast, followed by a 24-hour blood sampling period postdose. Dersimelagon 300 mg was administered once daily from day 6 to day 14 with coadministration of rosuvastatin 10 mg on day 10 and pravastatin 40 mg on day 14. Blood samples to determine plasma concentrations of rosuvastatin and pravastatin were collected from day 10 to day 15 to evaluate the effect of dersimelagon on the PK profiles of rosuvastatin and pravastatin. To determine dersimelagon plasma concentrations, blood samples were collected just prior to daily dosing of dersimelagon from day 7 to day 9 and up to 24 hours postdose on day 10 and day 14.

To determine the concentrations of rosuvastatin and pravastatin, plasma samples were prepared by solid phase extraction and analyzed using validated LC/MS/MS method with an LLOQ of 0.2 ng/mL (rosuvastatin) or 0.5 ng/mL (pravastatin). In both cases, deuterium-substituted compound of the analyte was used as the internal standard. Detection was performed using multiple reaction monitoring. Accuracy and coefficient of variation on sample analyses for all analytes met the prespecified acceptance criteria.

**Part 4: Verapamil**

Participants received a single oral dose of dersimelagon 100 mg on the morning of day 1 after an overnight fast, followed by a 48-hour blood sampling period postdose. Verapamil 80 mg was administered 3 times daily from day 5 to day 10 with coadministration of dersimelagon 100 mg on day 9. Blood samples were collected to determine the plasma concentration of dersimelagon from day 9 to day 11 to evaluate the effect of verapamil on the PK profile of dersimelagon. The bioanalytical methods for dersimelagon were described in the Methods for part 1.

# Supplementary Results

## Supplementary Table 1. Summary of potential inhibition of dersimelagon to CYPs

| **CYPs** | **Direct Inhibition** | | **Time-dependent Inhibition** | | **AUCR^b^**  **300 mg Dersi** |
| --- | --- | --- | --- | --- | --- |
|  | **IC_50_ (µmol/L)** | **R_1_ and R****_1,gut_^a^**  **300 mg Dersi** | **Shifted IC_50_ (µmol/L)** | **R_2_ ^a^**  **300 mg Dersi** |  |
| CYP1A2 | >100 | 1.00 (R_1_) | 72.2 | NC | NC |
| CYP2B6 | 48.9 | 1.00 (R_1_) | 36.3 | NC | NC |
| CYP2C8 | 24.2 | 1.01 (R_1_) | 13.9 | NC | NC |
| CYP2C9 | 9.16  5.13 µmol/L (K_i_) | 1.01 (R_1_) | 8.30 | NC | NC |
| CYP2C19 | 23.4 | 1.01 (R_1_) | 28.1 | NC | NC |
| CYP2D6 | >100 | 1.00 (R_1_) | >100 | NC | NC |
| CYP3A (substrate: midazolam) | 74.6 | 1.00 (R_1_)  75 (R_1,gut_) | 23.1  64.1 µmol/L (K_I_)  0.0141 min^-1^ (k_inact_) | 8.96 | 2.37 |
| CYP3A (substrate: testosterone) | 89.5 | 1.00 (R_1_)  63 (R_1,gut_) | 30.6 | NC | NC |
| UGT1A1 | 1.45  1.19µmol/L (K_i_) | 1.04 (R_1_) | NC | NC | NC |
| UGT1A3 | 22.6 | 1.00 (R_1_) | NC | NC | NC |
| UGT2B1 | >50 | 1.00 (R_1_) | NC | NC | NC |

^a^: R_1_, R_1,gut_, and R_2_ were calculated from the basic models described in the DDI guidelines,^11^ ^b^: AUCR was calculated from the MSPK model described in the DDI guidelines.^11^

Arithmetic mean C_max_ at steady state (1730 ng/mL for dersimelagon 300 mg) were estimated by population PK model analysis for dersimelagon.

Key parameters of dersimelagon for the MSPK model, fu,p: 0.017, Rb: 0.60, Fa: 1, Ka: 0.469

Cut-off: R1 <1.02, R2 <1.25, 0.8≦AUCR≦1.25

AUCR, area under the concentration-time curve ratio; IC_50_, half maximal inhibitory concentration; NC: not calculated;

Dersi, dersimelagon.

## Supplementary Table 2. Summary of potential inhibition of dersimelagon to drug transporters

| **Transporters** | **Cut-off** | **IC_50_ (µmol/L)** | **I_gut_/IC_50_ or I_inlet, max,u_/IC_50_ or I_max,u_/IC_50_**  **300 mg Dersi** |
| --- | --- | --- | --- |
| P-gp | I_gut_/IC_50_<10 | 0.349 | 5088 |
| BCRP | I_gut_/IC_50_<10 | 0.467 | 3803 |
| OATP1B1^a^ | I_inlet, max,u_/IC_50_<0.1 | 0.158 | 0.68 |
| OATP1B3^a^ | I_inlet, max,u_/IC_50_<0.1 | 0.0471 | 2.29 |
| OAT1 | I_max,u_/IC_50_<0.1 | 71.2 | 0.00 |
| OAT3 | I_max,u_/IC_50_<0.1 | 0.227 | 0.192 |
| OCT2 | I_max,u_/IC_50_<0.1 | >28.7 | 0.00 |
| MATE1 | I_max,u_/IC_50_<0.02 | 5.64 | 0.01 |
| MATE2-K | I_max,u_/IC_50_<0.02 | >27.4 | 0.00 |

^a^: IC_50_ values when incubated with 30-minute pre-incubation.

I_gut_/IC_50_, I_inlet,max,u_/IC_50_, and I_max,u_/IC_50_ were calculated from the equations described in the DDI guidelines.^11^

Arithmetic mean C_max_ at steady state (1730 ng/mL for dersimelagon 300 mg) was estimated by population PK model analysis for dersimelagon.

Key parameters of dersimelagon for the calculation of I_inlet,max,u_ and I_max,u_, fu,p: 0.017, Rb: 0.60, Fa: 1, Ka: 0.469

Dersi, dersimelagon; IC_50_, half maximal inhibitory concentration.

## Supplementary Table 3. Summary of pharmacokinetic parameters and drug-drug interaction effects for metabolites of reference drugs (PK population)

|  | **Part 1**  **1-hydroxy midazolam** | | **Part 1**  **4-hydroxy midazolam** | | **Part 2**  **o-hydroxy atorvastatin** | | **Part 2**  **p-hydroxy atorvastatin** | | **Part 2**  **β-hydroxy simvastatin** | |  |
| --- | --- | --- | --- | --- | --- | --- | --- | --- | --- | --- | --- |
| **Parameter** | **Alone**  **(N = 34)** | **+ Dersi**  **(N = 33)** | **Alone**  **(N = 34)** | **+ Dersi**  **(N = 33)** | **Alone**  **(N = 28)** | **+ Dersi**  **(N = 34)** | **Alone**  **(N = 33)** | **+ Dersi**  **(N = 34)** | **Alone**  **(N = 33)** | **+ Dersi**  **(N = 28)** | |
| C_max_ | | | | | | | | | | | |
| Geometric mean (CV%) | 4.10  (47.2) | 5.06  (51.8) | 0.50  (32.9) | 0.53  (33.9) | 0.35  (83.1) | 1.09  (87.8) | 11.09 (55.0) | 22.65 (60.2) | 2.52  (59.3) | 7.21  (76.0) | |
| LS mean | 4.10 | 5.06 | 0.50 | 0.53 | 0.35 | 1.09 | 11.09 | 22.65 | 2.52 | 7.30 | |
| LS mean ratio (90% CI) | 123.3  (108.3–140.4) | | 106.9  (100.5–113.6) | | 309.3  (243.4–393.2) | | 204.3  (167.0–250.0) | | 289.1  (251.9–331.6) | | |
| AUC_0-∞_ | | | | | | | | | | | |
| Geometric mean (CV%) | 11.3  (39.0) | 14.1  (29.5) | 1.56  (29.1) | 1.74  (24.0) | 10.5  (40.4) | 18.2  (65.5) | 98.0  (41.7) | 187.0  (54.6) | 31.2  (69.3) | 75.2  (80.1) | |
| LS mean | 11.3 | 14.2 | 1.56 | 1.74 | 10.5 | 18.1 | 98.0 | 187.0 | 32.0 | 70.2 | |
| LS mean ratio (90% CI) | 125.7  (118.1–133.8) | | 111.2  (106.4–116.3) | | 172.9  (125.5–238.1) | | 190.5  (172.8–210.1) | | 219.3  (186.5–257.9) | | |

Data are presented as geometric mean (C_max_: ng/mL, AUC_0-∞_: ng‧hr/mL) (geometric CV%), LS mean (C_max_: ng/mL, AUC_0-∞_: ng‧hr/mL), and LS mean ratio (expressed as a percentage) (90% CI) with and without the perpetrator drug. Doses for each reference drug were 2, 40, and 40 mg for midazolam, atorvastatin, and simvastatin, respectively. Dersimelagon doses were 300 mg.

AUC_0-∞,_ area under the plasma concentration–time curve from time zero to infinity; C_max_, maximum observed plasma concentration; CV, coefficient of variation; Dersi, dersimelagon; LS, least squares; PK, pharmacokinetic.

**Figure Legend**

**Supplementary Figure 1**. Mean plasma concentration-time profiles of (A) 1-hydroxy midazolam, (B) 4-hydroxy midazolam, (C) o-hydroxy atorvastatin, (D) p-hydroxy atorvastatin, and (E) β-hydroxy simvastatin with and without dersimelagon on semi-logarithmic scales (main figures) and linear scales (inserts). Data in inserts are displayed as mean + standard deviation. h, hours.
^a^n = 31 at 0.25 h. ^b^n = 32 at pre-dose. ^c^n = 27 at 0.25 h and 48 h. n = 26 at 24 h. n = 24 at 5h. ^d^n=27 at 0.25h and 48h. n=26 at pre-dose. ^e^n=27 at 0.5h and 48h. n=26 at pre-dose, 0.25h, and 24h. n=25 at 5h.. ^f^n=27 at 2h. n=26 at 1.5h. n=24 at 1h. n=22 at 0.5h. ^g^n =24 at 5 h.


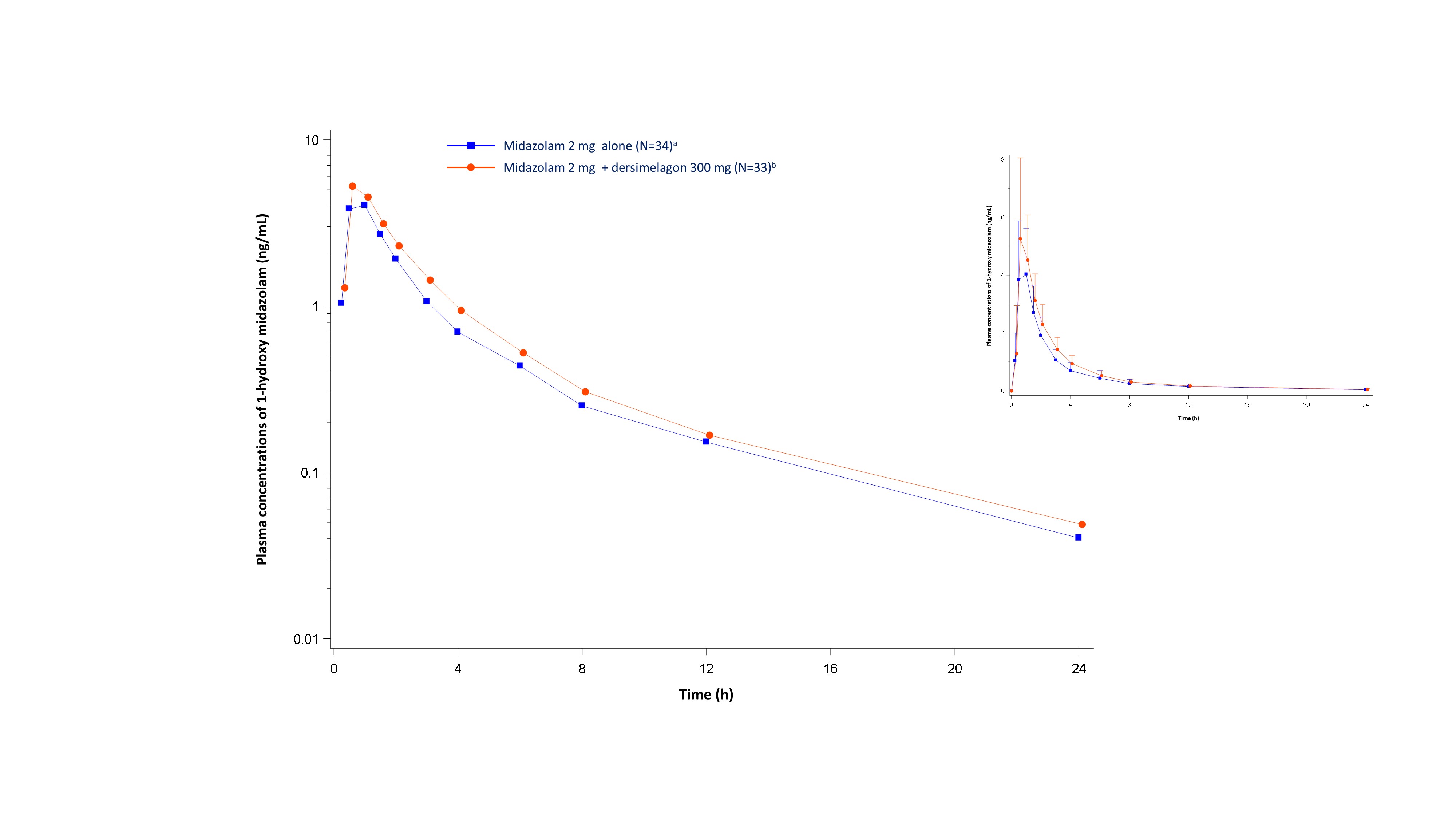
**A**

**B**


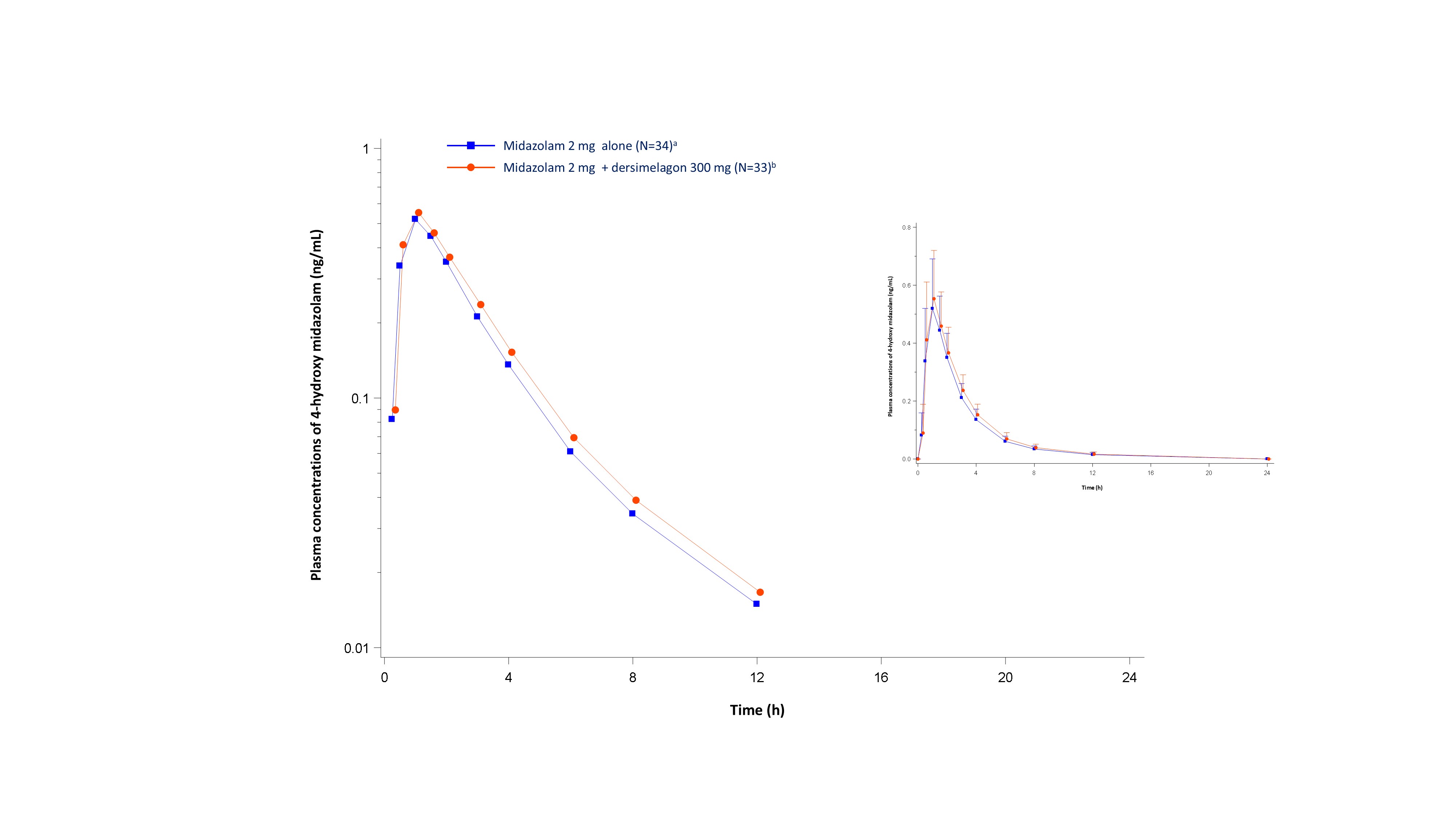


**C**

**
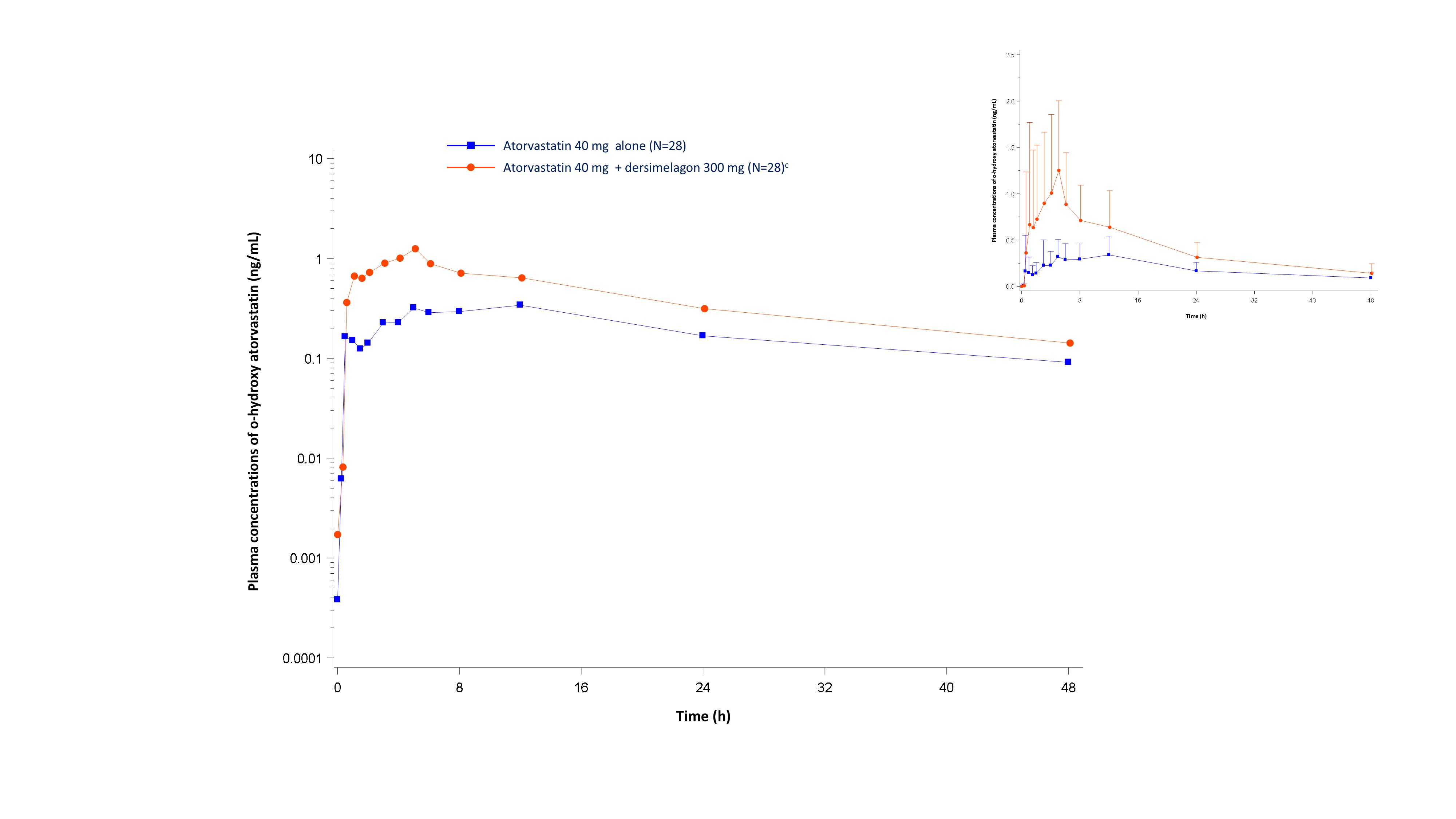
**

**D**

**
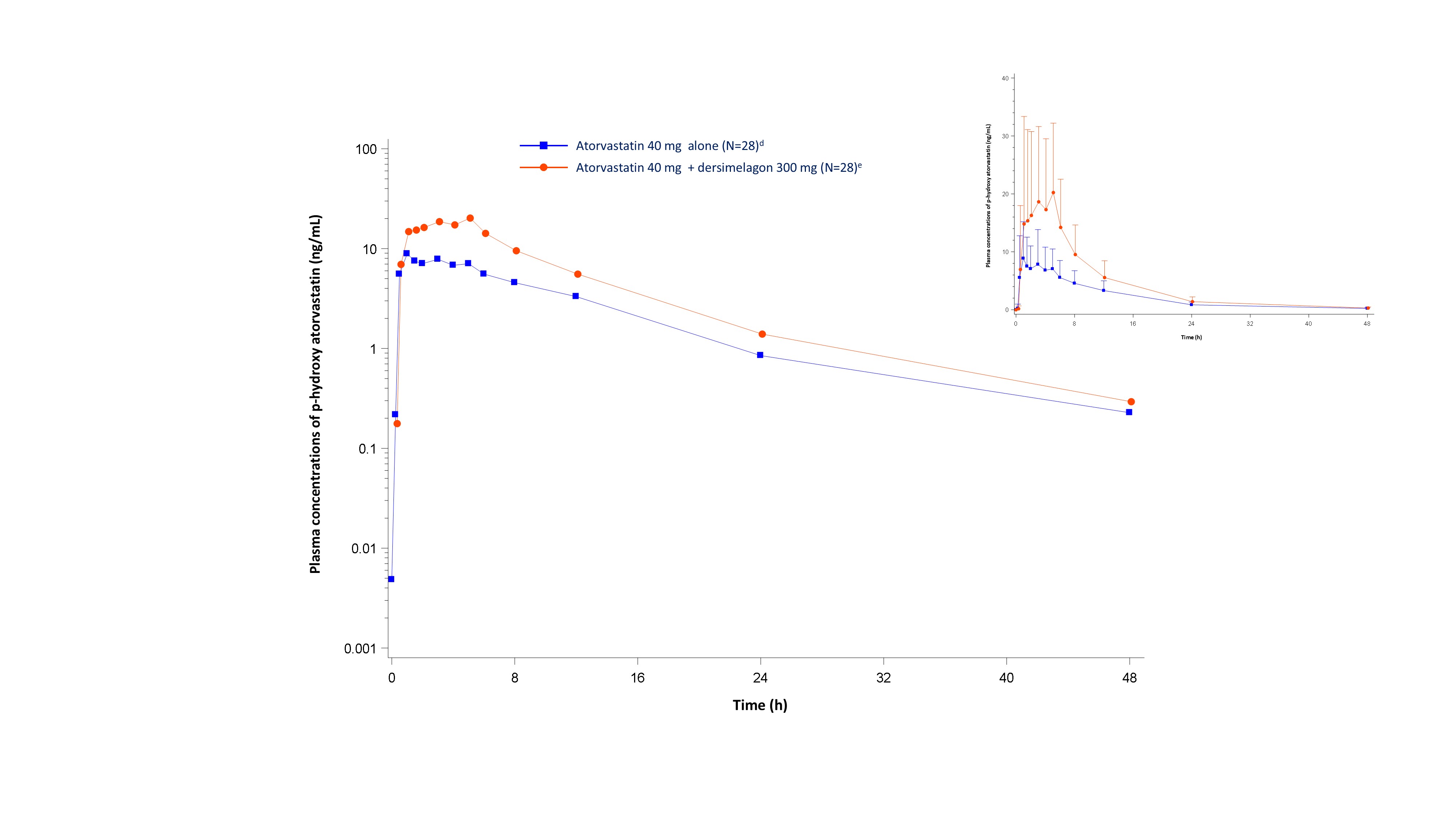
**

**E**


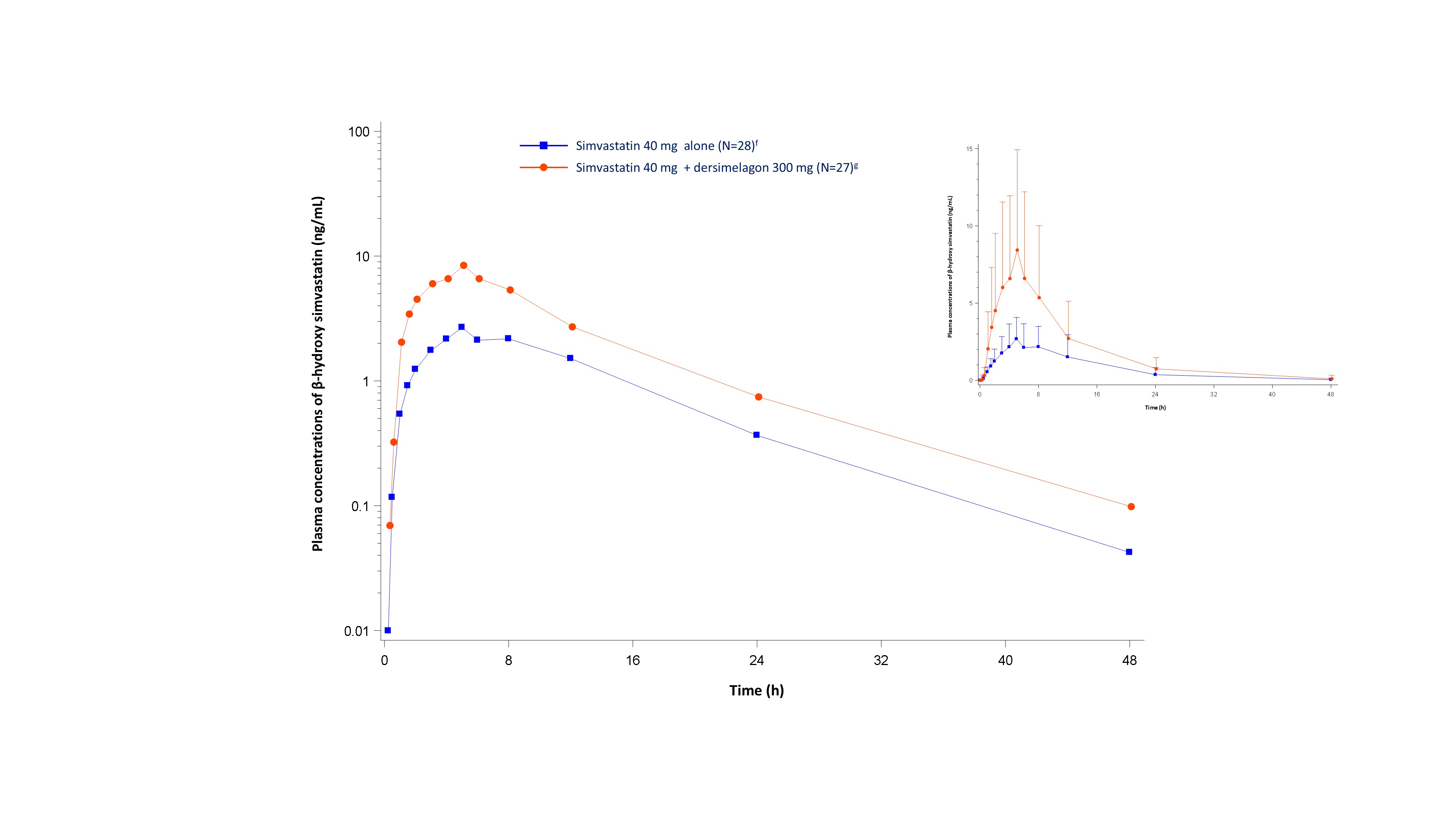

Supplement: Supplementary file 1 — Data S1. [file PRP2-13-e70069-s001.docx]
